# Development of a point-of-care-device for fast detection of periodontal pathogens

**DOI:** 10.1186/s12903-015-0155-y

**Published:** 2015-12-24

**Authors:** Cornelia Gaertig, Katja Niemann, Jana Berthold, Lisa Giel, Nadine Leitschuh, Christoph Boehm, Liudmila Roussak, Katja Vetter, Dirk Kuhlmeier

**Affiliations:** Nanotechnology Unit, Department of Diagnostics, Fraunhofer Institute for Cell Therapy and Immunology, Perlickstraße 1, 04103 Leipzig, Germany; Friedrich Schiller University, Jena, Germany; Scanbec GmbH, Bitterfeld-Wolfen, Germany

**Keywords:** Bacterial quantification, Periodontitis, Real-time PCR, Microbial diagnostics, Lyophilization

## Abstract

**Background:**

A number of pathogens can cause severe destruction of the periodontal apparatus during the course of periodontitis. The aim of this work was the development of a diagnostic device for the use at the point-of-need for the detection of periodontal pathogens to enable a personalized therapy for treatment of periodontitis.

**Methods:**

This test system is based on the polymerase chain reaction of DNA isolated from periodontal pathogens and was examined to precisely detect species-specific sequences on a rotating chip with lyophilized reagents for polymerase chain reaction. The preservation of the reagents was optimized to ensure their stability during the storage.

**Results:**

In the current work, we have developed a model point-of-care device and showed a proof of concept. It requires low sample volume, is timesaving and can therefore facilitate early diagnosis and treatment of periodontal diseases.

**Conclusions:**

The developed device can provide fast diagnosis of the composition and amount of patients’ oral flora and might help to assess the stage of periodontitis infection. This can facilitate an optimization of therapeutic approaches in order to prevent some of the more serious consequences of the disease.

**Electronic supplementary material:**

The online version of this article (doi:10.1186/s12903-015-0155-y) contains supplementary material, which is available to authorized users.

## Background

Approximately 90 % of the world’s population experience dental or oral problems during their lifetimes [1]. 11.2 % of adults are affected by severe periodontitis, thus making it a pertinent topic in the dental health care sector [2].

Periodontitis is a complex inflammatory response of the human body against microorganisms, which can lead to severe tissue destruction in the oral cavity [3].

Risk factors leading to infections have been identified as smoking, diabetes, occurrence of special microorganisms, obesity, psychological and hygienic aspects. Furthermore, genetic factors, age, gender and ethnicity might also play a role in the development of periodontal disease [4].

For the diagnosis of periodontitis, several practices have been established. Direct periodontal examinations include patients’ anamnesis, measurements of probing pocket depth and clinical attachment level, and taking dental radiographs. Additionally, genetic susceptibility tests or examination of the subgingival microflora and gingival crevicular fluid are optional methods to evaluate periodontal disease [5]. Depending on the results of the medical diagnosis and stage of the disease, an appropriate therapy follows. Approaches such as the optimization of oral hygiene, regular professional cleaning, scaling/debridement and root planing, the use of antibiotics for controlling bacterial plaque formation and inflammation, and even in extreme cases surgery can be necessary to prevent tooth loss [6].

Of the approximately 600–700 bacterial species that are living in subgingival areas, only a few of them are associated with periodontitis and can lead to significant changes in oral microbial community, quantity and composition [7, 8]. In order to assess the stage of periodontitis infections and initiate effective treatments, it is helpful to determine both the identities of the interacting species as well as the overall number of pathogens living in periodontal pockets. The work presented here focuses on bacteria species shown to be associated with periodontitis, which can be delineated into three classes based on pathogenicity: *E. corrodens, F. nucleatum, P. micra* are slightly pathogenic, *P. intermedia, C. rectus, E. nodatum* are moderately pathogenic and *A. actinomycetemcommitans, P. gingivalis, T. denticola* strongly pathogenic organisms [9]. Despite the fact that further species, the expression of typical biomarkers and the individual lifestyle of people can influence the development of disease as well, the detection of these marker species can be a first step in the process of identifying a disease or the medical condition in more detail.

If the types of bacterial species living in periodontal pockets can be identified, this information can be used to better classify the nature and severity of infection and tailor a personalized therapy best suited to reversing its course [10].

Only a few point-of care devices for nucleic acid based detection have been developed and established on the market so far. Cepheid’s GeneXpert, for example, is an automated device for polymerase chain reaction (PCR) with special sample preparation cartridges for tuberculosis pathogens, MRSA or group B streptococci. However, this high-cost system does not enable the option to implement several multiplexed reactions in one test run. IQuum recently presented the PCR-driven analysis of HIV in 1.5 h using the LIAT analyzer and Lutz et al*.* demonstrated a lab-on-a-foil system realizing an amplification reaction for MRSA in less than 20 minutes [11]. Furthermore, Van Oordt et al. [12] reported in 2012 about an integrated system for nucleic acid detection or immunoassays with the help of a disposable Labdisk platform.

In the present study, the development of a simple to use, low-cost lab-on-chip system for the detection, identification and quantification of periodontal bacteria is the primary consideration in the design of the molecular biological assay and diagnostic device. The diagnostic system consists of two major elements: a microfluidic cartridge, which contains all necessary PCR reagents in a dehydrated format, and a hardware device responsible for driving and controlling the work-flow. The latter is a radially-divided heating block assembly containing multiple stable temperature zones for the different steps of the PCR, combined with a centrally located rotary motor containing a mounting for a polycarbonate (PC) PCR chip. This chip harbors the patient sample in reaction chambers and rotates through the temperature zones on the heating block in order to carry out the PCR. The rising amplicon amount can be detected via fluorescence measurements.

Conceptually similar rotation devices exist, such as the Focus 3M™ Integrated Cycler, which uses the combination of centrifugal force and infrared energy as heating source for the PCR. Sample analysis is realized either in a disposable universal disc for user assays or for particular diseases in a chip with specific kits requiring many pipetting steps or cooling of reagents [13]. Also Sun et al*.* [14] showed 2007 a circular microchip for amplification applying ferrofluids and magnetic forces, revealing the problem of low sample throughput.

The lab-on-a-chip based amplification and measurement system presented in this work has the potential to optimize conventional periodontitis diagnostics in the future by minimizing laboratory handling steps and enable fast realization of PCR, avoiding a long shipping time of patient samples. Furthermore, the chip-based setup can be integrated with modules for DNA isolation in later versions. In this way, we aim to eliminate time-consuming and labor-intensive steps during sample preparation.

## Methods

### Bacterial strains/ cultivation conditions/ DNA isolation

Periodontitis-causing bacteria were obtained from the German collection of Microorganisms and Cell Cultures (DSMZ, Braunschweig, Germany) and grown in appropriate medium (see Additional file 1: Table S1). Colonies or bacteria pelleted from liquid media by centrifugation could be picked for DNA isolation. DNA purification was accomplished with the help of the QIAamp DNA Mini Kit® (Qiagen, Hilden, Germany) according to the manufacturer’s instructions. DNA yield and purity were measured with the NanoDrop instrument 1000 (Thermo Scientific, Wilmington, USA).

### PCR

The detection and quantification of periodontal bacteria amplification were carried out using a Lightcycler 480 II. Primer sequences are given in Additional file 2: Table S2. The primers for amplification of the species-specific DNA fragment were designed during this project with the help of the Primer3 software. Universal 16S primers were adopted from Kommedal et al. [15].

Cycling conditions for the amplification were as follows: 5 minutes of initial incubation at 95 °C, followed by 40 cycles of amplification (95 °C for 10 s, 51 °C for 10 s and 72 °C for 15 s). After each PCR run, an additional melting curve analysis was carried out to characterize the produced DNA molecules.

### Standard curves

For establishing standard curves, dilution series of bacterial DNA ranging from 2 to 2 million genome equivalents were used in a four-fold attempt for quantitative real-time PCR. Standard curves were created by default with the fit points method of the Lightcycler programme. For the overall bacterial count, *Porphyromonas gingivalis* served as a representative for 16S PCR with an average copy number of four 16S gene copies. The standard was set up to achieve an estimated pathogen number for quantification. It is known that several copies of the 16S gene can occur within one prokaryotic genome, with a maximum of 15 copies for *Bacillus thuringiensis*. This fact makes it difficult to estimate a realistic overall bacterial count in a diverse population of bacteria with strongly varying copy numbers between the individual species. The periodontitis-causing organisms used in our investigation are believed to be in the lower copy number range according to information given in the Ribosomal RNA Operon Copy Number Database [16]. To prove this, the unknown copy number for *E. corrodens* was determined during the course of this work. For unsequenced organisms, copy number determination can be achieved with the help of Southern Blot analysis. A 16S probe can detect the number of equivalent regions in the restricted genome, as previously shown by Lee et al. [17].

### Southern blot

To determine the 16S copy number of *E. corrodens* via Southern Blot, approximately 10 μg DNA were digested with restriction enzymes *Eco*RI HF, *Hind*III (New England Biolabs, Ipswich, USA), *Nco*I, *Pst*I, *Xba*I (Jena Bioscience GmbH, Jena, Germany*) and Xho*I FD (Thermo Fisher Scientific, Waltham, USA) at 37 °C overnight. The samples, along with the 16S PCR amplicon as positive control, were separated on a 1 % agarose gel at 4 °C. The gel was prepared for capillary blot with depurination (0.25 M HCl), denaturation (1.5 M NaCl, 0.5 M NaOH) and neutralization (3 M NaCl, 0.5 M Tris-HCl), followed by overnight blotting onto a nylon membrane.

For hybridization, the Biotin PCR Labeling Master (Jena Bioscience, Jena, Germany) was used to generate a probe. 16S primers and template DNA from *E. corrodens* were applied to produce a biotinylated amplicon by incorporation of biotinylated dUTPs during PCR. The probe was purified with the NucleoSpin® Gel and PCR Clean-up Kit (Macherey-Nagel, Düren, Germany). To ensure a complete target sequence for hybridization, an examination of possible cutting sites within the sequence was conducted: an enzymatic incubation of the 16S amplicon with the aforementioned enzymes and subsequent check by comparing the band size of digested amplicon to the control DNA in an agarose gel was carried out.

After pre-hybridization overnight at room temperature followed by a 2-hour incubation at 42 °C, hybridization with 200 ng probe/ml buffer was carried out. Hybridization and detection procedures were performed as described in the Biotin Chromogenic Detection Kit (Thermo Scientific, Schwerte, Germany), resulting in a coloured precipitate.

### PCR-on-a-chip

The PCR was implemented on 1 mm thick PC chips with cavities that hold a volume of 10 μl of reaction mix. The chips were sealed with sealing foils (nerbe plus GmbH, Winsen/Luhe, Germany) from both sides with the PCR mix embedded.

**Fig. 1:**
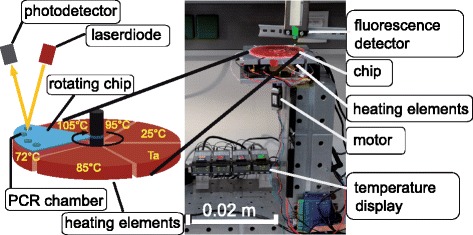
Thermocycling device with different temperature zones and a rotating PCR chip. Left: A schematic drawing illustrates the arrangement of the heating elements (Ta = annealing temperature) and the fluorescence detector. A rotating chip passes each zone and at 72 °C the fluorescence signal is measured. The right image shows the lab set-up, including the driving motor and temperature control

For amplification, the PCR chip rotates on the thermocycling device, which is shown in Fig. 1. Briefly, it is composed of six circularly arranged pie-slice-shaped heating blocks, three of them for accomplishing denaturation, annealing and elongation steps and three additional blocks for achieving rapid temperature changes inside the cavities.

For proof of concept of the apparatus, a well-established PCR of *S. aureus* (ATCC 2592), with an amplicon size of 279 bp was used. Cycling conditions were an initial 5 min denaturation, followed by 30 s at each of the sequential 95, 61 and 72 °C steps for amplification for a total of 40 cycles, with 5 s spent on the plates located between each of the three.

The reaction mix itself was optimized with several additives as described in the following ‘lyophilization section’ in order to increase the PCR-compatibility of PC and to enable lyophilization of the mix.

### Lyophilization

A test to determine the effect of lyophilization of PCR reagents upon the amplification reaction was conducted using the *E. corrodens* specific PCR as well-characterized test system. The PCR mix contained a final concentration of 0.25 μM for each primer, 50 % 2x SBYR Green Mastermix (Roche, Mannheim, Germany), 5 % trehalose, 0.25 μg/μl BSA, 0.75 % PEG 8000, 10 ng DNA and the remainder ddH_2_O. The lyophilization mix was frozen at -80 °C for 15 min and finally freeze-dried for three hours in a precooled lyophilizer. Long-time storage up to four months at 4 °C and room temperature were compared, with mixes protected from light, both with and without the addition of polymerase stabilizing trehalose, using the Lightcycler system for real-time PCR. A negative control was run during lyophilization to exclude any contamination during the process.

## Results

### Standard curves

**Fig. 2:**
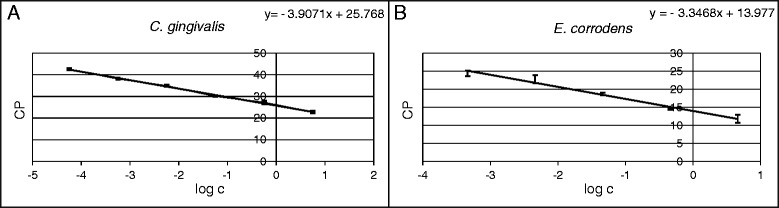
Standard curves: crossing point (CP) versus sample concentration. **a** C. gingivalis with an efficiency of 1.80 ranging from 20 to 2 million bacteria, and (**b**) E. corrodens with an efficiency of 1.99 ranging from 200 to 2 million bacteria. All data are presented as means ± SD

For the detection and quantification of the ten periodontitis-causing bacteria used in this study, and to determine the detection limits, quantitative PCR assays were established. By utilizing dilution series of bacterial genome copy numbers, standard curves were created according to the resulting amplification curves. As an example, the standard curves of *C. gingivalis* and *E. corrodens* are given in Fig. 2. To obtain reliable and reproducible results, the efficiency of the PCR should be greater than 1.8. Standard curves were set up for the remaining periodontal bacteria in the same way.

Detection limits determined by the Lightcycler 480 II were as follows: *P. intermedia* 2000 copies/reaction, *C. gingivalis* 20 copies/reaction, *C. rectus* 2 copies/reaction, *A. actinomycetemcommitans* 10 copies/reaction, *P. gingivalis* 10 copies/reaction, *E. corrodens* 200 copies/reaction, *P. micra* 2000 bacteria, *T. denticola* 20000 copies/reaction, overall count 200 copies/reaction.

### Southern Blot

**Fig. 3:**
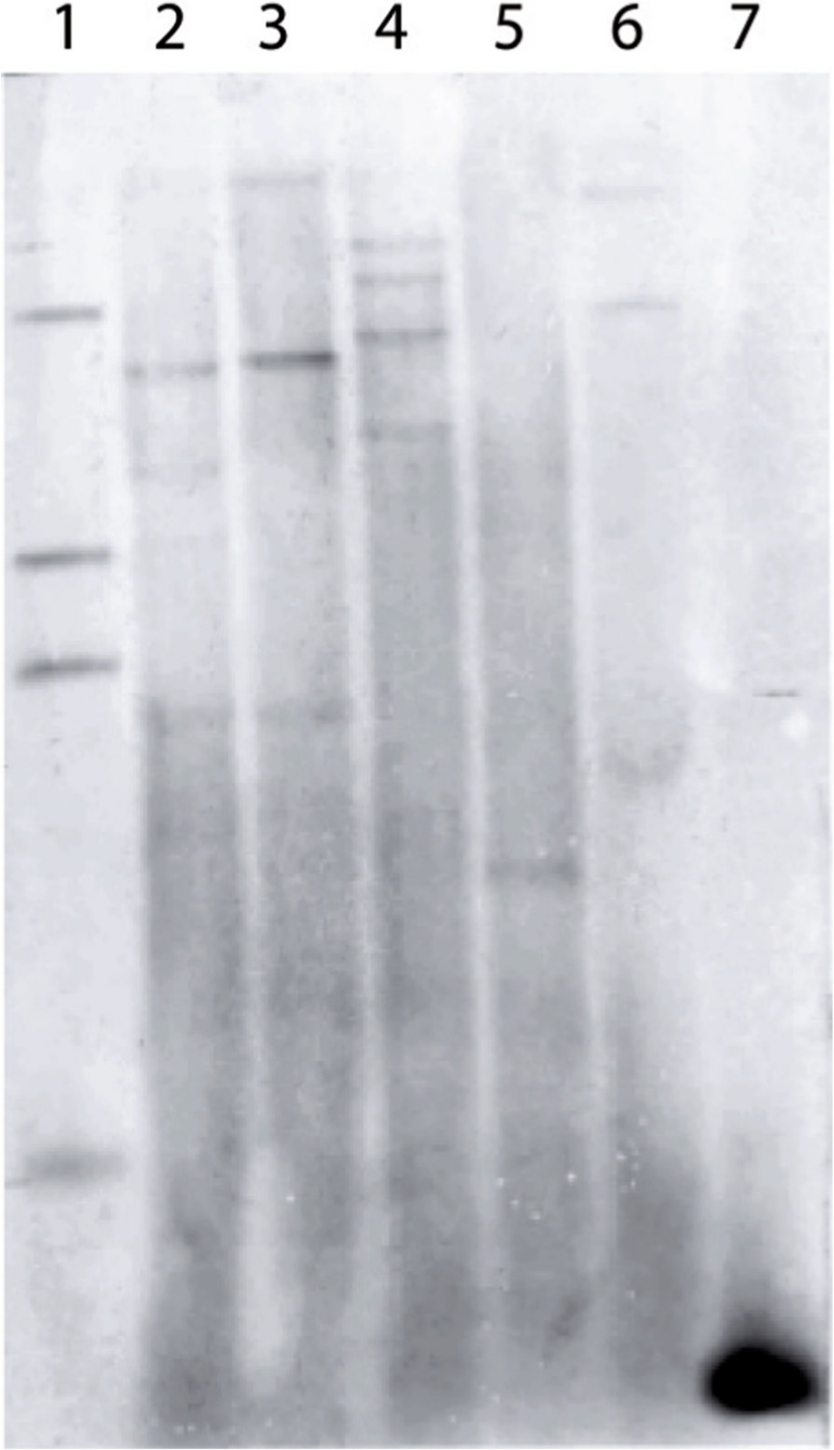
16S copy number of E. corrodens determined by Southern hybridization. Genomic DNA was cut with 1) *Eco*RI, 2) *Hind*III, 3) *Nco*I, 4) *Pst*I, 5) *Xba*I, 6) *Xho*I. 7) positive control. *Eco*RI, *Hind*III and *Pst*I show the maximum number of 4 bands

The restriction endonucleases *Eco*RI, *Hind*III, *Nco*I, *Pst*I, *Xba*I *and Xho*I were used to estimate the number of 16S operons present in the genome of *E. corrodens.* These enzymes were additionally tested to assure that there was no cutting site within the 16S region, where the detection probe hybridizes during the Southern Blot, in order to prevent occurrence of too many bands resulting from binding of the probe to cut target sequences. As shown in Fig. 3, a maximum number of four bands could be detected after cutting with *Eco*RI, *Hind*III and *Pst*I. The presence of fewer visible bands for the restriction with *Nco*I, *Xba*I *and Xho*I could be the result of the emergence of long DNA strands containing two or more 16S regions after enzymatic incubation. According to our investigations, a 16S operon number of four is very likely for *E. corrodens*, therefore using *P. gingivalis*, which contains the same 16S operon number, as the standard for estimating the overall bacterial count, seems to be very appropriate.

### PCR-on-a-chip

First fluorescence measurements were performed with the Qiagen ESElog fluorescence measurement system placed directly above the 72 °C temperature zone of the thermocycling device, which is responsible for the elongation step of the PCR. The ESElog USB E470/D520 is suitable for detection of the DNA-intercalating SYBR Green dye contained in the PCR mastermix.

**Fig. 4:**
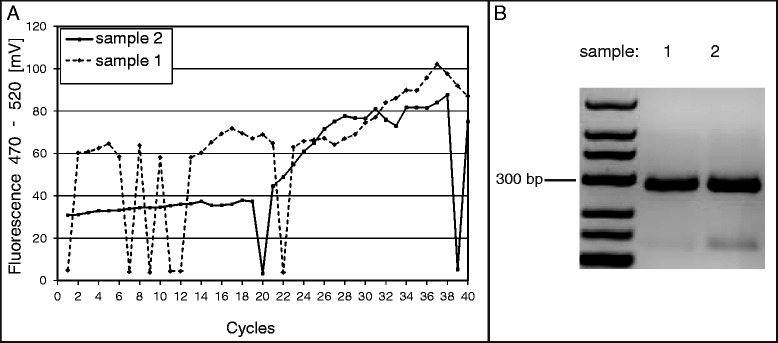
Real-time fluorescence detection. Singleplex PCR shown on the lab-on-chip system. **a** Fluorescence measurement exemplarily shown for two samples. **b** Resulting amplicon on a 2 % agarose gel

For every cycle during which the sample-containing chip passed through each of the different temperature zones on the thermocycling device, the continuous rise in fluorescence corresponding to the amount of *S. aureus*-specific amplicon present at that time was measured. The resulting rise in fluorescence as measured over 40 cycles by the detector can be seen in Fig. 4.

During the heating steps, bubble formation in the PCR chambers led to aberrations in the course of amplification as exemplarily shown in cycle 20 and 39 of sample 2 (see Fig. 4a). Nevertheless, the expected increase of fluorescence and a positive PCR result of the targeted 279 bp region of *S. aureus* were demonstrated in the initial tests (see Fig. 4b).

### Lyophilization

**Fig. 5:**
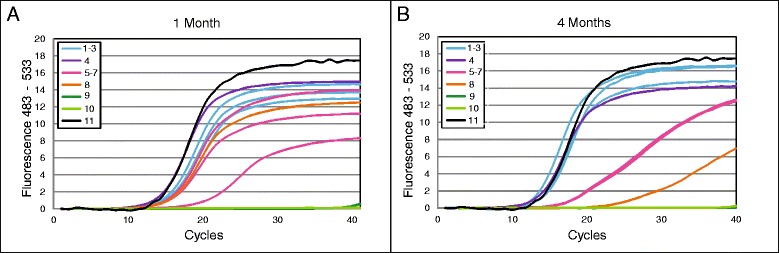
Lyophilization test. Comparison of the polymerase activity after 1 (**a**) and 4 (**b**) months of storage of freeze-dried PCR mixes.1-3) storage at 4 °C, 4) storage at 4 °C without addition of trehalose, 5-7) storage at room temperature, 8) storage at room temperature without addition of trehalose, 9) PCR negative control, 10) negative control stored at 4 °C, 11) positive control after lyophilization

Preservation of the biological activity of the polymerase after freeze-drying was demonstrated for the examined period of four months. *E. corrodens*-specific PCR evaluation shows that this long-term storage at 4 °C after lyophilization did not lead to any changes of the amplification curve, whereas storage at room temperature flattened the typical course of the curve and its characteristic exponential increase. In this case, the real-time PCR crossing points occurred in a delayed manner (see Fig. 5 and Additional file 3: Table S3).

The addition of trehalose to the PCR mix proved to be essential for long-term storage, especially at room temperature. Amplification with the reaction mix without trehalose led to a poor signal, lower overall fluorescence levels and delayed CPs after storage at room temperature for four months.

## Discussion and conclusions

Periodontitis is a ubiquitous disease worldwide with the need for effective diagnostics, fast treatment and inhibition of its progression in order to prevent tooth loss. Furthermore, oral infections can potentially lead to severe secondary diseases such as sepsis, systemic or cardiovascular disorder and pneumonia [18].

In order to facilitate the detection and quantification of bacteria species indicative of the disease, we developed a PCR-driven point-of-care device for periodontitis-associated pathogen identification and showed basic functionality.

The quantitative PCR assay for bacterial strains was developed successfully and standard curves were set up with the Lightcycler system. For absolute quantification, unknown concentrations of samples can be compared with already established standard curves, where at least one single standard sample of known concentration falling within the range of the imported curve has to be included in each PCR run to serve as a reference. Ximénez-Fyvie et al*.* already showed in 2000 that subgingival plaque samples of healthy patients often contain significantly lower bacterial numbers, so the achieved detection limits here of as few as two bacteria in some cases are suitable to analyze the often very high bacterial loads in cases of existing periodontitis [19]. Developing the device further, the described quantification procedure will be transferred onto the point-of-care device to enable testing of patient-derived samples in the future.

Southern Blot analysis could identify four copies of the 16S region in the genome of *E. corrodens*. This method circumvents sequencing of the complete *E. corrodens* genome, whose full sequence is not available yet. However, this number might be higher in the case of incomplete cutting by the restriction enzymes or in the case of two or more 16S areas being located on one DNA fragment arising from restriction digest. Nevertheless, these results show that using *P. gingivalis* as a representative for periodontopathogenic bacteria is appropriate for generating the standard curve for overall bacterial counts with a typical 16S copy number of four. The use of this bacterium for the generation of a universal standard curve for the assessment of total microbial counts was already described by Kirakodu et al*.* [20].

As a proof of concept, the PCR assay could be shown to work in PCR chips rotating over several heating zones on a specially designed thermocycling device. Due to the small reaction volume required and the lack of necessity for heating and cooling steps by the heating blocks themselves, the reaction time was one-third faster than in conventional thermocyclers, even without any extra care taken to further optimize the individual PCR steps.

In the future, this reaction will be optimized with shortened denaturation/annealing steps and PCR-additives or fast polymerases, for example a KAPA2G Fast DNA Polymerase (Kapabiosystems, Wilmington, USA) with an elongation time of approximately 1 s/kb, thus leading to a significantly shortened overall reaction time. Moreover, a further reduction of the reaction volume can accelerate the heat transfer, consequently lowering the required amount of reagents and costs [21].

In our point-of-care system, pipetting steps for individual reagents are minimized leading to fewer sources of error and increasing the convenience for the end user. Therefore, the use of lyophilized reaction mixes was tested in order to enable long-term storage. It was demonstrated that *Taq* polymerase could be protected against degradation and loss of inactivity when stored at 4 °C, while some reduced activity was evident when stored at room temperature for up to four months. Based on these results the reaction mix is suitable for storage in conventional refrigerators.

However, long-term studies to examine the shelf life of the reaction mixes are currently being carried out in order to see whether *Taq* is damaged after a longer period of time following the dehydration process.

The optical system for fluorescence detection was integrated onto the device, revealing the problem of bubble formation within the sample during PCR. PCR mix degassing, higher glycerol content and changing hydrophobicity as suggested by Trung et al*.* and Jankowski et al*.* [22, 23] did not completely eliminate this problem and occasional aberrations in the course of fluorescence measurements were observed. Retrofitting of the system with a heating block or a hot air fan at the top of the chip with taller cavities might help to prevent evaporation and subsequent bubble formation.

In the further development of this device, an on-chip DNA purification module will be integrated into the chip and multiplex reactions with sequence-specific fluorescent probes should be established in order to avoid the use of unspecific intercalating dyes. Furthermore, biomarker tests could be implemented into the chip construction, which helps to characterize and optimize patients’ state of health as well.

In general, a significant problem for treatment and prevention of periodontitis is that, up to now, standard home dental care products such as toothbrushes and floss cannot completely eliminate bacterial plaque formation and prevent occurrence of the disease [9]. Therapeutic measures e.g. surgery, tooth extraction and eventually dentures are a massive financial burden for patients or dental insurance, if purchased. Taken together, this shows the importance of developing a quick to apply, reliable point-of-care system for the diagnosis and quantitative classification of periodontal infections in order to facilitate the immediate implementation of the optimal healthcare plan that is available and for the diagnosed stage of the disease. Socransky et al*.* [10] showed 1998 in their complex theory that there are typical early and late colonizers in the periodontal disease progression, indicating different manifestations of periodontitis requiring respective individualized treatment.

To find out about the microbial composition of subgingival patient samples, dentists usually have to send plaque samples to a clinical laboratory. Cultivation, immunological, or DNA probe analysis and recently more sensitive nucleic acid based test systems such as the microIDent® (Hain Lifescience, Nehren, Germany) can deliver information for optimal therapy, however still with the necessity and cost of shipment to laboratories [24].

The simple, model point-of-care system presented here, which can be easily applied in every dental office, reduces both time and costs for diagnostics and eliminates the necessity for transport to external laboratories and therefore facilitates crucial fast treatments in the future. Additionally, the chip can be adjusted and used for further applications in medical areas with a need for fast detection and analysis of pathogens, such as those causing sepsis or regional/global pandemics.

### Availability of supporting data

The supplemental data of Additional file 1: Table S1, Additional file 2: Table S2 and Additional file 3: Table S3 supporting the methods and results of this article are included within the additional Excel files.

## Additional files

Additional file 1: Table S1. Organisms and cultivation conditions. (XLSX 11 kb) Additional file 2: Table S2. Primer sequences and product size. (XLSX 12 kb) Additional file 3: Table S3. Crossing points (CP) corresponding to Fig. 5 (XLSX 10 kb)

## Abbreviations

BSA: Bovine serum albumin
CP: Crossing point
PCR: Polymerase chain reaction
PC: Polycarbonate
PEG: Polyethylene glycol
